# Impact of Thermal Pretreatment of Saliva on the RT-PCR Detection of SARS-CoV-2

**DOI:** 10.1155/2022/7442907

**Published:** 2022-06-01

**Authors:** Orlando Miguel Morais, Manuel Rui Azevedo Alves, Paulo Alexandre da Costa Fernandes

**Affiliations:** ^1^Escola Superior de Tecnologia e Gestão, Instituto Politécnico de Viana do Castelo, Rua Escola Industrial e Comercial de Nun'Álvares, Viana do Castelo 4900-347, Portugal; ^2^CISAS, Escola Superior de Tecnologia e Gestão, Instituto Politécnico de Viana do Castelo, Rua Escola Industrial e Comercial de Nun'Álvares, Viana do Castelo 4900-347, Portugal

## Abstract

The use of saliva directly as a specimen to detect viral RNA by RT-PCR has been tested for a long time as its advantages are relevant in terms of convenience and costs. However, as other body fluids, its proven inhibition effect on the amplification reaction can be troublesome and compromise its use in the detection of viral particles. The aim of the present work is to demonstrate that saliva pretreatment may influence the RT-PCR amplification of three gene targets of SARS-CoV-2 significantly. A pool of RNA from confirmed COVID-19 patients was used to test the influence of heat pretreatment of saliva samples at 95°C for 5, 10, 15 and 20 min on the amplification performance of ORF1ab, E, and N SARS-CoV-2 genes. Prolonged heating at 95°C significantly improves the Ct value shift, usually observed in the presence of saliva, increasing the limit of detection of viral genes ORF1ab, *E,* and *N*. When tested using a cohort of COVID-19 patients' saliva, the increased time of heat pretreatment resulted in a significant increase in the detection sensitivity.

## 1. Introduction

Saliva is a complex fluid composed of a mixture of components produced by major and minor salivary glands [1] and other constituents from the oral mucosa and microbiome [2, 3]. Its suitability as a specimen for biomonitoring [2] and to detect respiratory viruses has been tested over several years [4–6], and its use in routine testing and screening campaigns to detect SARS-CoV-2 has also been subjected to assessment [4, 7–16]. A recent meta-analysis using 25 published studies involving RT-PCR detection of SARS-CoV-2 in saliva samples highlighted considerable discrepancies in reported findings, pointing out that many of these discrepancies could probably be attributed to experimental procedures, such as target populations, sample collection, and saliva processing protocols [17].

The US Food and Drug Administration has recently issued an emergency use authorization (EUA) for the so-called SalivaDirect assay, a method using saliva directly without extraction in the RT-PCRs [18]. However, the inhibition potential of saliva has been described in the literature [19] and has been a relevant obstacle to more widespread use of this specimen in diagnostic tests. Saliva, as other body fluids, is known to have constituents that may inhibit the RT-PCR [19]. In fact, RT-PCR is a very sensitive and powerful technique based on the amplification of small amounts of nucleic acids present in a sample. However, due to its enzymatic nature and complex mixture of components including fluorophores and oligonucleotides, several substances may affect the reaction mainly by interfering with the DNA polymerase activity/stability, nucleotide/nucleic acid stability, and fluorescence intensity [20, 21].

In the present work, saliva is used directly without extraction in RT-PCRs to detect 3 viral targets of SARS-CoV-2, and it is demonstrated that the inhibition potential of this matrix can be significantly influenced by the time of pretreatment at 95°C.

## 2. Materials and Methods

### 2.1. Sample Collection

The saliva samples from healthy individuals were collected under supervision by just letting mouth-accumulated saliva drop into empty sterile sputum containers.

Saliva samples from COVID-19 patients were collected as previously described [22] under supervision of a healthcare worker. All donors were previously informed in writing about the purpose and procedure of the study and consented to participate by providing the samples.

All samples were stored at 4°C until processing (not exceeding a maximum of 6 h) and then kept at −80°C until further use.

### 2.2. Preparation of Saliva Samples Prior to RT-PCR

Saliva samples for studies involving spiking were prepared to be used directly in the RT-PCR using a 20 mg × ml^−1^ proteinase K solution (NZYTech, Portugal), vortexing for 1 min, and heating at 95°C for 5 min as described by Vogels and colleagues [18] and also for 10, 15, and 20 min. Saliva samples from 23 COVID-19 patients were processed as described before and heated for 5 min at 95°C and 15 min at 95°C.

### 2.3. Real-Time Reverse Transcriptase-Polymerase Chain Reaction Assay (RT-PCR) for SARS-CoV-2

Samples were tested for SARS-CoV-2 using novel coronavirus (2019-nCoV) RT-PCR detection kit (Shanghai Fosun Long March Medical Science) and the CFX96 real-time PCR system (BioRad, Germany) in accordance with the manufacturer's instructions. A maximum of 10 *μ*l of saliva samples, prepared as previously described, were added to 20 *μ*l of the reaction mixture. Samples were then incubated at 50°C for 15 min and then at 95°C for 3 min, followed by 5 cycles at 95°C for 5 s and 60°C for 40 s and 40 cycles at 95°C for 5 s and 60°C for 40 s, targeting SARS-CoV-2 genes *N*, *E,* and ORF1ab. At the end of each of the last 40 cycles, the signals of FAM, JOE, and ROX were registered. A cycle threshold value (Ct value) less than or equal to 36 was defined as a positive test result, and more than 36 or no value was considered as negative.

### 2.4. Determination of the Limit of Detection (LOD)

RT-PCRs having 3, 30, and 300 copies/reaction were prepared using EDX SARS-CoV-2 Positive Run Control (Exact Diagnostics, BioRad, Germany) manufactured with synthetic RNA transcripts containing 5 gene targets (*E*, *N*, *S*, ORF1a, and RdRP genes of SARS-CoV-2, 200000 copies/ml each). Tests were performed in triplicate, and the repeatability of each assay was determined by analyzing the cycle threshold values (Ct) of parallel reactions and respective standard deviations (SD).

### 2.5. Real-Time Reverse Transcriptase-Polymerase Chain Reaction Assay (RT-PCR) in the Presence of Saliva Pretreated between 5 and 20 min at 95°C

A pool of positive SARS-CoV-2 RNA, extracted from nasopharyngeal swab samples from confirmed COVID-19 patients, was serial diluted (1 : 10 and 1 : 100) with RNase-free water (PanReac AppliChem, Germany). RT-PCR amplification reactions were performed in duplicate, as previously described, in the absence of saliva and in the presence of 6 *μ*l of saliva (negative for the presence of SARS-CoV-2) pretreated for 5, 10, 15, and 20 min at 95°C.

### 2.6. Statistical Analysis

Data were analyzed following conventional methodologies [23], mainly as available in the *R* base package [24], with a special reference to the “aov” and “TukeyHSD” functions to carry out ANOVA and post hoc tests for comparisons between the groups, “ad.test” for analysis of normality, and “wilcox.test” to perform Wilcoxon rank-sum tests with matched pairs for determination of significance of median differences. All graphs were produced using routines written by the authors using the *R* language. Excel was used as a general data organization and analysis tool.

## 3. Results and Discussion

### 3.1. Temperature Effect on the Inhibition Power of Saliva

Some published works comparing saliva and nasopharyngeal swabs (NPS) from the same COVID-19 patients demonstrate that an increase in the Ct values obtained for different viral targets occurs when saliva is used [22, 25]. According to previous works on saliva and its effects when used on PCR reactions, it is known that inhibitory effects can be reduced by extraction with Chelex-100 (in the case of parotid saliva) or just by heating in boiling water for at least 10 minutes in the case of whole mouth saliva (WMS) [19]. In the present work, saliva used directly in the RT-PCRs was prepared following a method previously described [18] and authorized for emergency use by the FDA, but introducing one modification, 95°C heating time, originally designed to inactivate the proteinase K used in the assay. This was done taking into consideration the results previously presented in relation to the heating effect on reducing the inhibition power of saliva in traditional PCR reactions [19].

**Figure 1:**
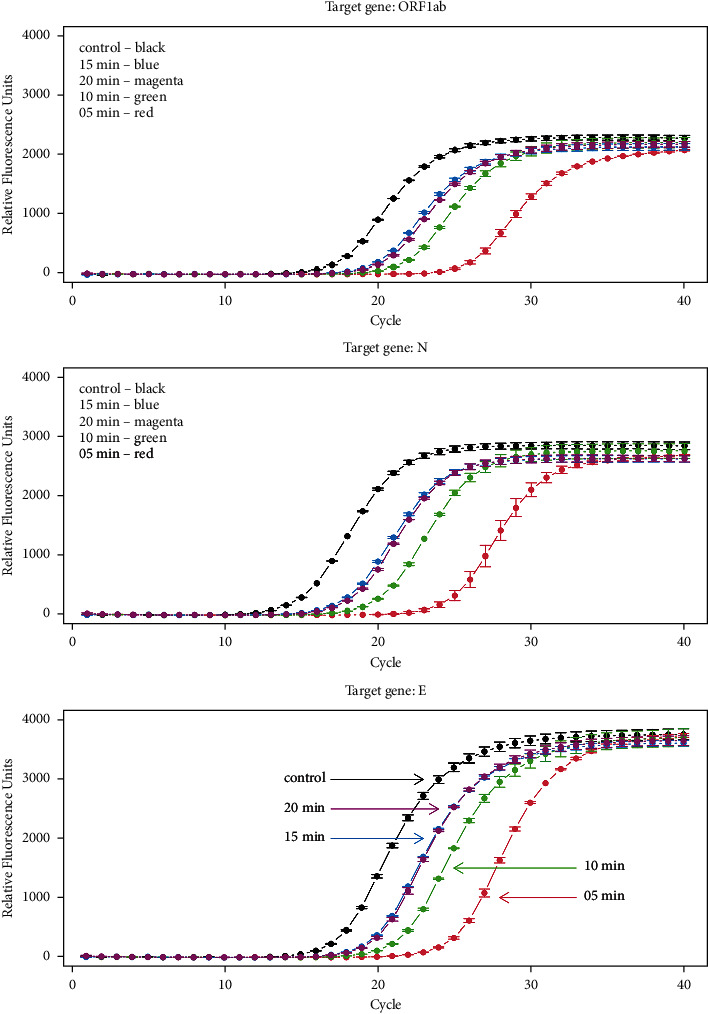
Amplification curves of a pool of RNA from COVID-19-positive patients in the presence of saliva pretreated at 95°C for 5 (red), 10 (green), 15 (blue), and 20 minutes (magenta). The control curve is shown in black. Curves correspond to a 1 : 10 dilution of the RNA pool.

The effect of saliva pretreated using different conditions on the performance of the RT-PCRs was therefore analyzed, namely, heating at 95°C during four different time periods: 5, 10, 15, and 20 min. After spiking the heat pretreated saliva samples with the same amount of viral RNA (a pool of RNA extracted and purified from COVID-19 patients), RT-PCR was performed and the corresponding amplification curves are presented in Figure 1.

As it can be clearly observed in Figure 1, which represents the amplification reactions of the 1 : 10 diluted pool of SARS-CoV-2 RNA, the curves are displaced to the right in the presence of saliva in relation to the control reaction (without saliva), but the magnitude of this displacement depends on the pretreatment time.

Curves obtained with saliva pretreated at 95°C for 5 min showed the highest displacement, corresponding to the worst performance. In average, and considering all the target genes and initial concentration of RNA, an increase of 7.87 ± 1.16 Ct values was observed in relation to the control reaction. On the other hand, heating for 15 min reduced the ΔCt values to just 2.27 ± 0.38 Ct in relation to control. As it is shown in Figure 1, curves of saliva pretreated for 15 and 20 min are practically superimposed. Finally, heating for an intermediate time of 10 min leads to an intermediate result. It can, therefore, be concluded that increasing heating time pretreatment to 15 min improves the reaction and prolonging heating for more than 15 min does not seem to have significant influence on the reduction of the inhibitory power.

**Figure 2:**
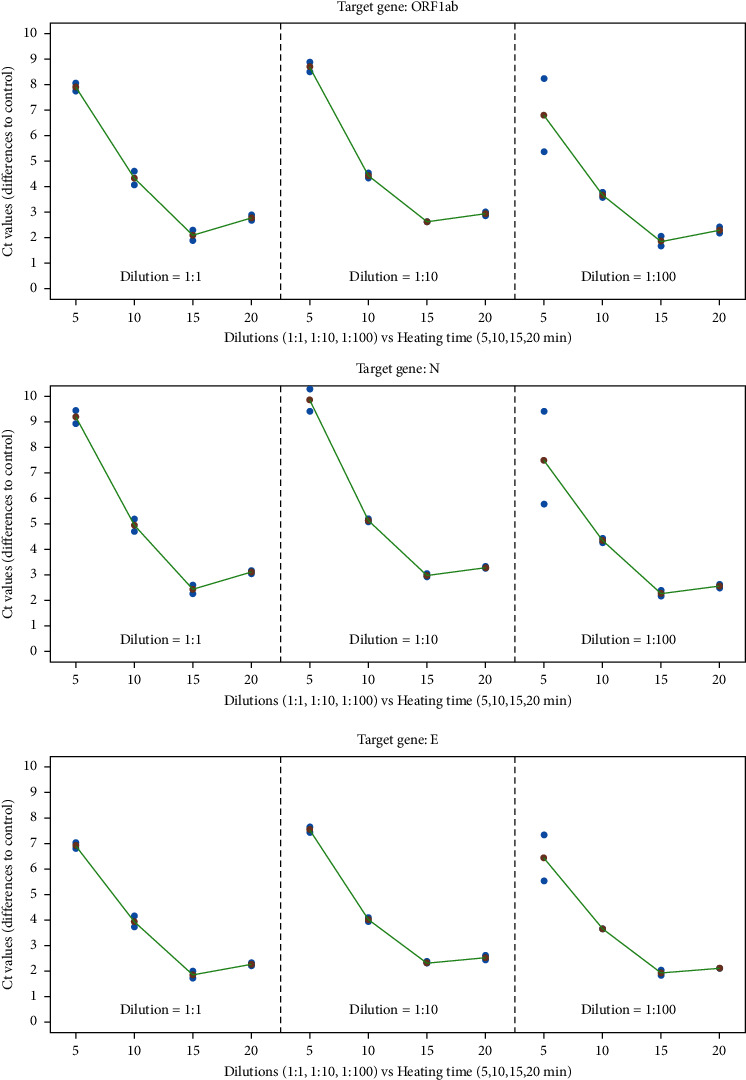
ΔCt values in relation to the control reaction and influencing factors: genes ORF1ab, *N,* and *E,* three dilutions (1 : 1, 1 : 10, and 1 : 100), and four heating times (5, 10, 15, and 20 min). Blue points are minimum and maximum differences to control, red points are mean values, and green lines are the joining mean values to improve interpretation.

Differences in Ct values according to heating time and dilution factors can be observed in Figure 2 and in Tables 1 and 2 with the results of two-way ANOVAs with dilution, heating times, and respective interactions as the main factors, as well as replications, carried out for each gene. Figure 2 clearly shows that increasing heating times leads to an improvement (decrease) in Ct values for genes ORF1ab, *N,* and *F*, while the dilutions (amount of initial RNA) used in this work have a much smaller impact on Ct values. The two-way ANOVAs, whose main results can be seen in Table 1, show that differences in Ct values caused by dilution are small but significant, while differences caused by heating time are highly significant. Checking differences with post hoc Tukey HSD tests (Table 2), it is seen that for all genes, the dilution 1 : 100 is significantly different from the others, with decrease in the magnitude in the order of 1, 1.1, and 0.57 Ct, respectively, for genes ORF1ab, *N,* and *E*, in comparison to the dilution 1 : 10.

In what concerns the heating time factor, the same Tukey HSD tests (Table 2) show that the decrease in Ct values ranges between 3 and 4 if heating time increases from 5 to 10 min and decreases around 2 Ct values more when time increases from 10 to 15 min.

As it can be seen in Table 1, differences due to replications or interactions are not significant.

It is worth noting that according to comparisons based on statistical approaches, there is no significant difference between heating pretreatments for 15 and 20 min. However, comparing Figures 1 and 2, heating for 20 min seems to be worse than heating for 15 min since all 20 min curves are slightly displaced to the right of 15 min curves in Figure 1. At the same time, the last point (20 min) in all displays presented in Figure 2 also shows a slight increase in Ct values in comparison to the 15 min points. These observations are true for all genes and for all dilutions. Therefore, although not supported by statistical evidence in face of the experimental design used, it seems that pretreatment involving heating for 15 min at 95°C is probably the best procedure.

Saliva, if treated for just 5 min, shows a greater inhibition power. This fact is reflected in a positive shift in the Ct values and also this has some clear effect on the reproducibility of the results, as the range of results is much wider when lower RNA concentrations are used, and this is particularly relevant when the target is gene N (Supplementary Figure S1).

The direct use of a sample without extraction in an RT-PCR has also been tried for other matrices [26] using heat inactivation. For saliva, a previously published work tested heat shock for 10, 20, and 30 min prior to RT-PCR, and the Ct values obtained were comparable at all the conditions used, despite undetermined at 10 min for *N* and ORF1ab genes [27].

### 3.2. Between-Person Variability

As demonstrated, in this work, it was observed that heating at 95°C has a dramatic effect on the reduction of the saliva inhibition power. However, numerous factors can affect saliva composition, which may vary between different people and circumstances related to the sample collection [28]. Therefore, it was decided to test 10 saliva samples from different healthy individuals, collected under the same protocol and circumstances.

**Figure 3:**
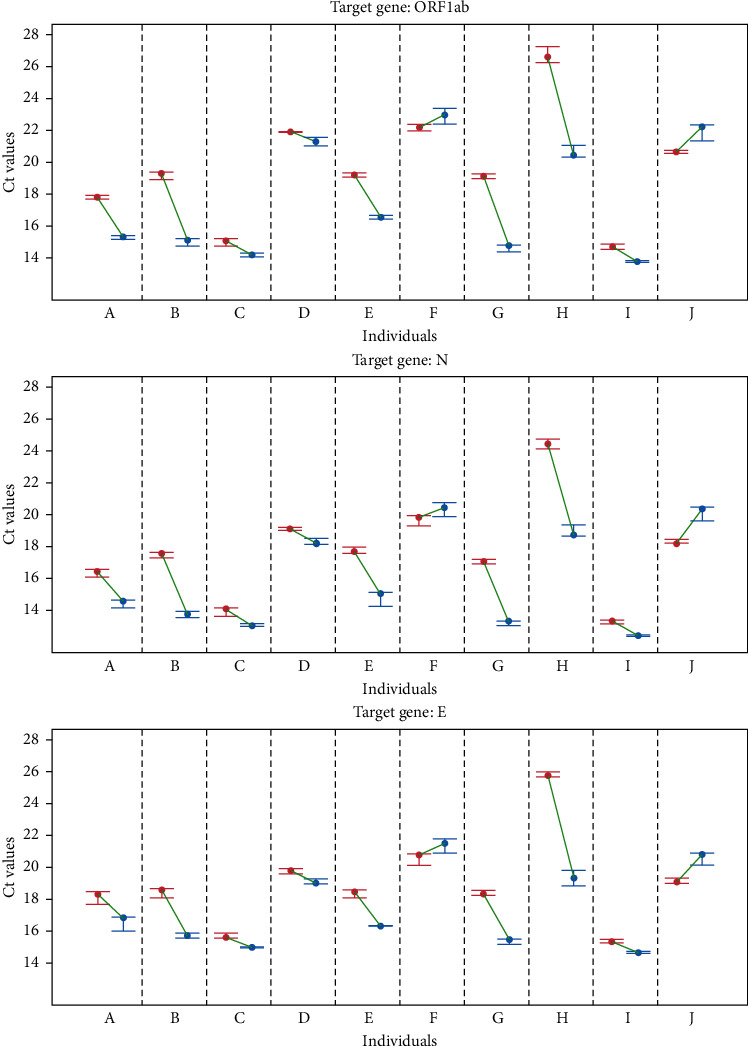
Minimum and maximum (bars) and median (points) Ct values after heating for 5 min (red) and 15 min (blue). Green segments represent variation in Ct median values due to differences in heating. Letters *A* to *J* refer to identification of 10 individuals.

All saliva samples were used in a RT-PCR with the same amount of viral RNA.  Figure 3 represents the variation of the Ct values between the reactions where saliva was heated at 95°C for just 5 min (as described in the FDA emergency use authorized protocol) and for 15 min (best conditions found in this work). As shown in Figure 3, the extension of pretreatment leads to a significant decrease in the Ct value of the RT-PCR. However, as it can be seen in Figure 3, the between-person variability is very high. In relation to the reaction without saliva, a median increase of 5.53 in the Ct values was obtained in the reactions performed in the presence of saliva heat treated for 5 min and 2.24 when the saliva was pretreated for 15 min. Therefore, although variable in magnitude, 80% of the saliva samples had their inhibitory power significantly reduced by just extending the heating time in the pretreatment step.

### 3.3. Influence of Saliva's Thermal Pretreatment on the Limit of Detection (LOD)

Considering that an increase in 3.32 Ct values is observed when the initial amount of template has a tenfold reduction, the high magnitude of the positive shift observed in the Ct values in the presence of saliva has significant consequences on the sensitivity of the method. The detection limit, as announced by the manufacturer (for nasopharyngeal swabs), is 300 copies/ml, corresponding to 3 copies/reaction. Thus, a test was carried out under different conditions of pretreatment of 6 *μ*l of saliva per reaction, and it was observed that the inhibitory effect has significant consequences for the LOD, although of different magnitudes for different viral targets. In fact, the detection limit announced by the manufacturer was only reached in the reaction without saliva and for two of the viral targets (genes *E* and *N*). When saliva is treated at 95°C for just 5 min, only gene *E* is detected, but 300 copies/reaction need to be present. After 15 minutes of treatment, the detection limit increases significantly and it is possible to detect 30 copies/reaction for gene *E* and 300 copies/reaction for genes N and ORF1ab. Considering that the criterion to consider a sample as positive for the presence of SARS-CoV-2 is to have amplification (Ct < 36) for at least two of the three targets (or just ORF1ab confirmed in a repetition), one should consider the most restrictive LOD when saliva is used, i.e., 5 × 10^4^ copies/ml of saliva.

Despite a significant decrease after heating saliva for 15 min at 95°C, its inhibition power still exists, indicating that other components, not thermolabile, may be present and interfere with the amplification reaction, although at a much lower level. Due to the high between-person variability, this effect should be regarded only as a rough estimation, obtained with a saliva sample presenting an inhibition power close to the median (in terms of ΔCt value).

The viral gene used as the target is of great importance as different targets show different sensitivities to the inhibition potential of saliva, and one must also consider that the heat-shock applied to the saliva before its use in RT-PCR may have influence on the integrity of the RNA present, leading potentially to a cleavage in smaller fragments [27] that may best suit the amplification of smaller amplicons.

### 3.4. Detection of SARS-CoV-2 Using Saliva of COVID-19 Patients

A total of 23 samples of saliva from COVID-19-confirmed patients (nasopharyngeal swab RT-PCR positive) were pretreated, as previously described, for 5 min and 15 min at 95°C. A detailed analysis of genes ORF, *N,* and *E* showed that the differences between the effects caused by the heating time are not normal, according to Anderson–Darling tests with *p* values lower than 0.003. Therefore, median values and interquartile ranges (IQRs) were calculated, and significance of the decrease in median values with increase in heating time was assessed with Wilcoxon rank-sum tests for paired samples. For gene ORFlab, the median decrease in Ct values was 0.61 (*p* < 0.0002 and IQR = 0.90), for gene N, the decrease was 0.68 (*p* < 0.0006 and IQR = 0.68), and for gene E, it was 0.45 (*p* < 0.0005 and IQR = 0.58). Despite an overall modest decrease in the Ct values when compared with the results obtained with saliva pretreated with heat prior to spiking, the results also show a high level of between-person variability which is also target dependent with ΔCt values ranging from −5.88 to +0.55 and, most importantly, a significant increase in the detection sensitivity. It was observed that with 5 min of pretreatment, only 20 samples were considered positive, while using a 15 min heating period, all 23 samples were considered positive, which corresponds to an increase in sensitivity in the range of 2.8% to 33.6%, with 13.0% as the most probable number (following a binomial test, *p* < 0.0005).

### 3.5. Final Remarks

This work has provided evidence that a simple modification of saliva thermal pretreatment might have very significant influence on the RT-PCR performance, reducing significantly the positive shift in the Ct value (of the three viral targets) of the reactions performed in the presence of saliva without extraction. In fact, the heat treatment at 95°C is not only needed to inactivate proteinase K, but it contributes to reduce the inhibitory power of saliva on the performance of the RT-PCR.

Even with the thermal pretreatment of 15 min, a certain degree of inhibition continues to occur causing a positive shift in the reaction Ct value. However, there is a significant improvement in the Ct value obtained with prolonged heating. The increased sensitivity that can be observed may make saliva a good candidate for use in SARS-CoV-2 screenings (and probably for other respiratory viruses) using commercial solutions widely available. Considering that a large part of contamination occurs through the transmission of small droplets of saliva, the increased sensitivity verified can make this specimen ideal for the detection of carriers with potential for the spread of respiratory viruses as SARS-CoV-2. Although the present study contributes to a better understanding of the effect of saliva used directly in RT-PCRs and focuses on how to minimize the respective inhibitory effects, this work clearly shows that a significant between-person variability exists. Therefore, it is important to extend the study with a wider number of samples and also analyze the within-person variability which was not addressed in the present work.

## Figures and Tables

**Table 1:** *p* values relative to dilution, heating time, replications, and interaction of dilution and heating time of two-way ANOVAs for genes ORF1ab, *N,* and *E*.

Factor	ORF	*N*	*E*
Dilution	0.0249	0.0313	0.0465
Heating time	3.4 × 10^−8^	5.49 × 10^−8^	8.83 × 10^−10^
Replications	0.4552	0.3768	0.5891
Dilution *∗* heating time	0.8058	0.6540	0.7964

**Table 2:** Differences in Ct values and respective *p* values, for factors dilution and heating time, for genes ORF1ab, N, and E, as determined by post hoc Tukey HSD analysis.

Factors		Gene ORF1ab		Gene *N*		Gene *E*	
		Ct difference	*p* value	Ct difference	*p* value	Ct difference	*p* value
Dilution	1 : 10–1 : 1	0.390	0.4569	0.558	0.5582	0.360	0.2144
	1 : 100–1 : 10	−1.013	0.0206	−1.138	0.0270	−0.566	0.0399
Heating time	10–5	−3.645	3.7 × 10^−6^	−4.030	7.2 × 10^−6^	−3.095	2.0 × 10^−7^
	15–10	−1.957	0.0011	−2.240	0.0014	−1.830	3.61 × 10^−5^
	20–15	0.487	0.5560	0.423	0.7609	0.263	0.6736

## Data Availability

All data are available upon request.

## Supplementary Materials

Supplementary Materials Figure S1 presents the amplification curves of a pool of RNA from COVID-19-positive patients in the presence of saliva pretreated at 95°C for different time periods.
