# Supplementary material for: Impact of Thermal Pretreatment of Saliva on the RT-PCR Detection of SARS-CoV-2
Source: Adv Virol. 2022 Jun 1;2022:7442907. doi: 10.1155/2022/7442907 (PMC9177321; doi:10.1155/2022/7442907)
Supplement: Supplementary Materials — Figure S1 presents the amplification curves of a pool of RNA from COVID-19-positive patients in the presence of saliva pretreated at 95°C for different time periods. [file 7442907.f1.pdf]

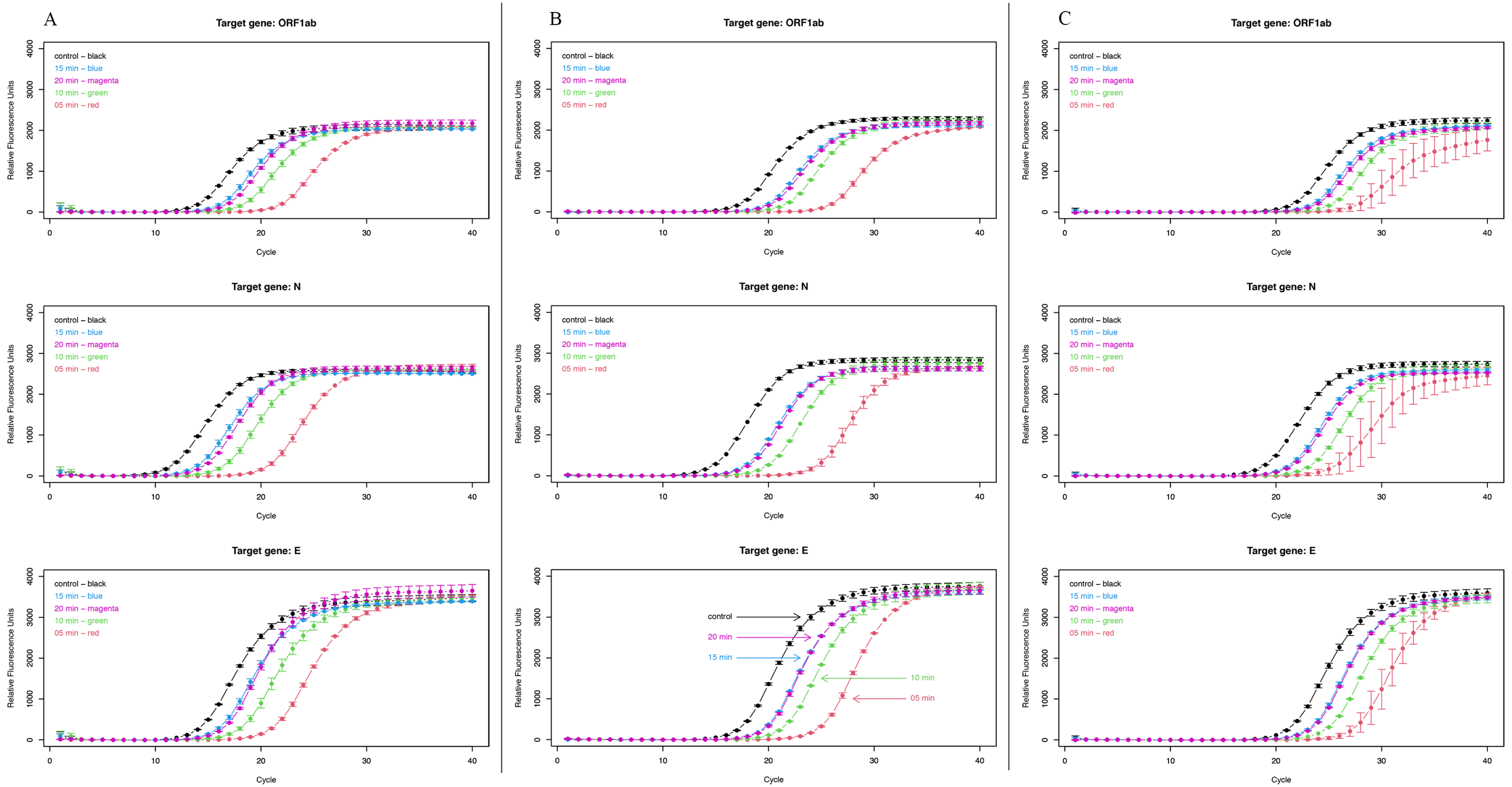

Figure S1. Amplification curves of a pool of RNA from positive COVID-19 patients in the presence of saliva pre-treated at 95ºC for 5 (red), 10 (green), 15 (blue) and 20 minutes (magenta). The control curve is shown in black. A. Undiluted RNA pool; B. 1:10 dilution of the RNA pool; C. 1:100 dilution of the RNA pool
